# Circ_0005526 contributes to interleukin-1β-induced chondrocyte injury in osteoarthritis via upregulating transcription factor 4 by interacting with miR-142-5p

**DOI:** 10.1080/21655979.2022.2048773

**Published:** 2022-03-24

**Authors:** Paerhati Wahafu, Aixian Xu, Bo Zhao, Yanan Tuo, Junkui Yang

**Affiliations:** a Department of Orthopedics, The Sixth Affiliated Hospital of Xinjiang Medical University, Urumqi, Xinjiang, China; b Department of Orthopedics District 1, The People’s Hospital of Zhaoyuan City, Zhaoyuan, Shandong, China; c Department of Orthopedics District 2, The People’s Hospital of Zhaoyuan City, Zhaoyuan, Shandong, China

**Keywords:** Circ_0005526, miR-142-5p, transcription factor 4, osteoarthritis, chondrocyte

## Abstract

Circular RNAs (circRNAs) can regulate the progression of osteoarthritis (OA) via serving as competing endogenous RNAs (ceRNAs). This work was performed for functional research of circ_0005526 in Interleukin-1β (IL-1β)-induced OA injury. Circ_0005526, microRNA-142-5p (miR-142-5p) or transcription factor 4 (TCF4) expression was measured via reverse transcription-quantitative polymerase chain reaction assay. Cell analysis was performed by Cell Counting Kit-8 assay for cell viability, EdU assay for cell proliferation and flow cytometry for cell apoptosis. The protein level detection was conducted using western blot. Target analysis was carried out via dual-luciferase reporter assay and RNA pull-down assay. Circ_0005526 was upregulated in OA cartilage tissues and IL-1β-exposed chondrocyte cells. IL-1β inhibited cell viability and proliferation but enhanced cell apoptosis and inflammation, then these damages were attenuated after downregulation of circ_0005526. Circ_0005526 interacted with miR-142-5p, and circ_0005526 knockdown suppressed IL-1β-induced OA progression through upregulating miR-142-5p. TCF4 was regulated by circ_0005526 via targeting miR-142-5p. The function of circ_0005526 was also achieved by upregulation of TCF4. These results unraveled that circ_0005526 promoted IL-1β-induced chondrocyte injury in OA via suppressing miR-142-5p binding to TCF4.

## Graphical Abstract

**Figure:**
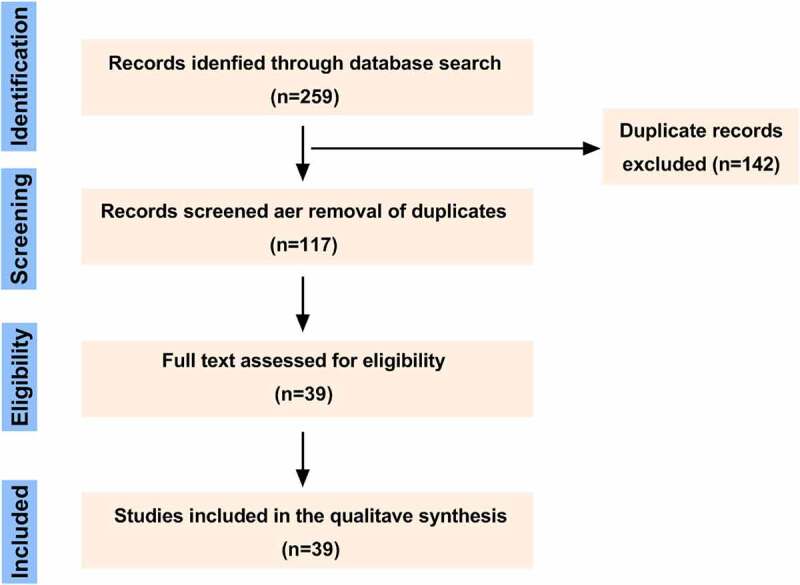

## Introduction

Osteoarthritis (OA) is a common joint disease with destruction of articular cartilages [1]. OA has seriously affected the life quality of human beings, and there is no effective strategy for treatment of OA [2]. Chondrocyte dysfunction is the leading cause of cartilage degeneration and OA progression [3]. The previous studies have indicated that the pathogenesis of OA is related to chondrocyte apoptosis and inflammatory response [4,5]. Interleukin-1β (IL-1β) signaling is a risk factor of OA, and IL-1β induction is commonly used to establish OA cell model [6]. Investigating the molecular mechanism in IL-1β-induced OA progression is important.

Circular RNAs (circRNAs) are known as competing endogenous RNAs (ceRNAs) to inhibit the binding between microRNAs (miRNAs) and downstream genes, consequently regulating the pathogenic development of human diseases [7]. Chen *et al*. uncovered that circRNA-9119 suppressed IL-1β-induced chondrocyte apoptosis via interacting with miR-26a to result in upregulation of phosphatase and tensin homolog (PTEN) [8]. Wu *et al*. elucidated that circ_0134111 aggravated inflammatory reaction and extracellular matrix degradation of chondrocytes in OA through downregulating suppressor of cytokine signaling 1 (SOCS1) by targeting miR-515-5p [9]. Circ_0005526 has been shown to function as miRNA sponges in OA [10], but the regulatory function of circ_0005526 remains unknown.

Small miRNAs also play central roles in OA progression via regulating gene levels [11]. MiR-142-5p can protect against OA progression by targeting small glutamine rich tetratricopeptide repeat containing beta (SGTB) or C-X-C chemokine receptor type 4 (CXCR4) [12,13]. Transcription factor 4 (TCF4) is considered as a apoptotic factor in human chondrocytes [14], and Wang *et al*. stated that miR-137 affected OA development by downregulating TCF4 [15]. The target relation between miR-142-5p and TCF4 is not clear. In addition, circular RNA IQ motif-containing GTPase-activating protein 1 (circ_IQGAP1) served as a miR-671-5p sponge to mediate TCF4 level in IL-1β-caused chondrocyte injury [16]. The regulation of circ_0005526 for TCF4 by targeting miR-142-5p has not been researched.

Herein, circ_0005526 was hypothesized to be a miR-142-5p sponge and TCF4 was assumed to be a downstream target for miR-142-5p. The goals of this study were to explore the biological function and regulatory mechanism of circ_0005526 in IL-1β-induced chondrocyte injury.

## Materials and methods

### Human tissues

Tissue acquisition was performed after informed consent files were provided by all participators. OA cartilage tissues (n = 39) and normal cartilage tissues (n = 21) were respectively collected from OA patients who underwent total knee arthroplasty and amputees who had no OA at the Sixth Affiliated Hospital of Xinjiang Medical University. The clinical data of OA patients were shown in Table 1. These samples were stored in liquid nitrogen until further use. Additionally, this research was approved by Ethics Committee of the Sixth Affiliated Hospital of Xinjiang Medical University.

**Table 1.** Clinical parameters of OA patients

Clinical parameter	Total (n = 39)
Age (years)	62.15 ± 10.65
BMI (kg/m^2^)	28.15 ± 3.11
Gender	
Male	46%
Female	54%
Index OA knee	
Right	49%
Left	51%
CRP, mean (mg/L)	13.9 ± 4.2
ESR, mean (range, mm/h)	26.8 ± 5.7

OA: Osteoarthritis; BMI: Body mass index

CRP: C-reactive protein; ESR: Erythrocyte sedimentation rate

### Cell culture and IL-1β treatment

Human normal chondrocyte cell line CHON-001 (BioVector NTCC Inc., Beijing, China) was cultured in 5% CO_2_ incubator at 37°C, using Dulbecco’s modified Eagle’s medium (Sigma, St. Louis, MO, USA) with the supplement of 10% fetal bovine serum and 1% penicillin/streptomycin (Gibco, Carlsbad, CA, USA). OA cell model was established by treatment with 10 ng/mL IL-1β (Sigma) in CHON-001 cells for 12 h.

### Cell transfection

Lipofectamine™ 3000 transfection kit (Invitrogen, Carlsbad, CA, USA) was exploited for transfection of oligonucleotides or plasmids in CHON-001 cells, following the manufacturer’s protocols. Small interfering (si) RNA of circ_0005526 (si-circ_0005526), siRNA negative control (si-NC), mimic and inhibitor of miR-142-5p or negative control (miR-142-5p/miR-NC, anti-miR-142-5p/anti-NC) were bought from RIBOBIO (Guangzhou, China). The pcDNA plasmid (Invitrogen) was cloned with TCF4 sequence to construct pcDNA-TCF4 (TCF4).

### Reverse transcription-quantitative polymerase chain reaction (RT-qPCR) assay

Tissues and cells were lysed with Trizol reagent (Invitrogen) for total RNA extraction. Then 2 μg RNA/sample was used for synthesis of complementary DNA (cDNA) through ReverTra Ace® qPCR RT Kit (Toyobo, Kita-Ku, Osaka, Japan), followed by quantification reaction via SYBR® Green Realtime PCR Master Mix (Toyobo) using specific primers. The sequences were shown in Table 2. For level normalization, beta-actin (β-actin) for circ_0005526/TCF4 and U6 for miR-142-5p were applied as internal references. Then, relative expression was calculated by data analysis via 2^−∆∆Ct^ method [17].

**Table 2.** Primer sequences used for RT-qPCR

Name	Primer sequences (5’-3’)
circ_0005526	Forward: GCTATCATTGGTAGAGGTGGACT Reverse: TCATACTGGGATGAGGAATGCG
miR-142-5p	Forward: GCCGAGCATAAAGTAGAAAG
	Reverse: CTCAACTGGTGTCGTGGAG
TCF4	Forward: CCTGGCTATGCAGGAATGTT Reverse: CAGGAGGCGTACAGGAAGAG
β-actin	Forward: GAGCTACGAGCTGCCTGAC
	Reverse: CCTAGAAGCATTTGCGGTGG
U6	Forward: TCGCTTCGGCAGCACATATAC Reverse: TATGGAACGCTTCACGAATTTG

### Cell viability assay

1 × 10^5^ cells were transplanted into 48-well plates, followed by IL-1β treatment and cell transfection. 48 h later, cells were added with 10 μL/well CCK-8 solution (Beyotime, Shanghai, China). The plates were performed with 2 h of incubation at 37°C, then absorbance at 450 nm was tested by a microplate reader. Cell viability = viable cells/total cells × 100%.

### Cell proliferation assay

Cell proliferation was evaluated by EdU-positive cells [18]. EdU Proliferation Kit (Beyotime) was used in this study. According to the user’s manuals, cells were incubated with 100 µL EdU solution and nuclei were stained with diamidinyl phenylindole (DAPI; Beyotime). The micrographs were taken by a fluorescence microscope (Olympus, Tokyo, Japan). Then, EdU-positive cells were counted as merged cells by EdU and DAPI staining.

### Cell apoptosis assay

Flow cytometry was used for detecting cell apoptosis [19]. Apoptotic cells were examined after staining by Annexin V-fluorescein isothiocyanate (Annexin V-FITC) and Propidium Iodide (PI) through Annexin V-FITC Apoptosis Detection Kit (Invitrogen). Firstly, cells were resuspended with 1 × Binding Buffer and cell suspension (5 × 10^4^ cells) was incubated with 5 μL Annexin V-FITC for 10 min. Subsequently, 10 μL/well PI was added for 5 min and cells were observed on the flow cytometer (BD Biosciences, San Diego, CA, USA). Apoptosis rate = (Annexin V+/PI- and Annexin V+/PI+) stained cells/total cells × 100%.

### Western blot

After protein extraction by Radioimmunoprecipitation assay buffer (Sigma), protein concentrations of samples were determined via BCA Protein Assay Kit (Beyotime). Then, protein analysis was conducted in accordance with the standard operations of western blot [20]. The primary antibodies (Abcam, Cambridge, MA, USA) were indicated as follows: Proliferation Cell Nuclear Antigen (PCNA; ab18197, 1:1000), Cyclin D1 (ab16663, 1:1000), B-cell lymphoma-2 (Bcl2; ab32124, 1:1000), Bcl2-associated X (Bax; ab182733, 1:1000), Interleukin-6 (IL-6; ab233706, 1:1000), Tumor Necrosis Factor-α (TNF-α; ab183218), TCF4 (ab130014, 1:1000), β-actin (ab213262, 1:3000). Goat Anti-rabbit IgG, HRP-linked Secondary Antibody (Abcam, ab205718, 1:5000) was incubated for 1 h. The blots were visualized through Electrochemiluminescence Reagent (Sigma), followed by level analysis via Image J software (NIH, Bethesda, MD, USA).

### Dual-luciferase reporter assay

Interaction analysis was performed using dual-luciferase reporter assay [21]. The wild-type (WT) sequences of circ_0005526 and TCF4 3ʹUTR were inserted into pmirGLO luciferase plasmid (Promega, Madison, WI, USA), respectively. The constructed plasmids containing miR-142-5p binding sites were named as circ_0005526-WT and TCF4 3ʹUTR-WT. Then miR-142-5p binding sites in circ_0005526 and TCF4 sequences were mutated, and mutant-type (MUT) plasmids (circ_0005526-MUT and TCF4 3ʹUTR-MUT) were used as negative controls. After co-transfection with each plasmid and miR-142-5p or miR-NC for 48 h, luciferase intensity was analyzed through Dual-Luciferase Reporter Detection Kit (E1910; Promega).

### RNA pull-down assay

CHON-001 cells were transfected with Biotin-coupled miR-142-5p (Bio-miR-142-5p; RIBOBIO) and negative control (Bio-miR-NC; RIBOBIO) for 48 h. After incubation with streptavidin magnetic beads (Thermo Fisher Scientific, Waltham, MA, USA), the binding RNA was isolated and circ_0005526 was quantified using RT-qPCR.

### Statistical analysis

The experiments were independently repeated by three times with three parallels (n = 3). Data were shown as mean ± standard deviation and processed using SPSS 22.0 (SPSS Inc., Chicago, IL, USA), then Student’s *t*-test or analysis of variance (ANOVA) followed by Tukey’s test was exploited to analyze the difference. *P* < 0.05 demonstrated that difference was significant.

## Results

### Circ_0005526 was upregulated in OA

**Figure 1.**
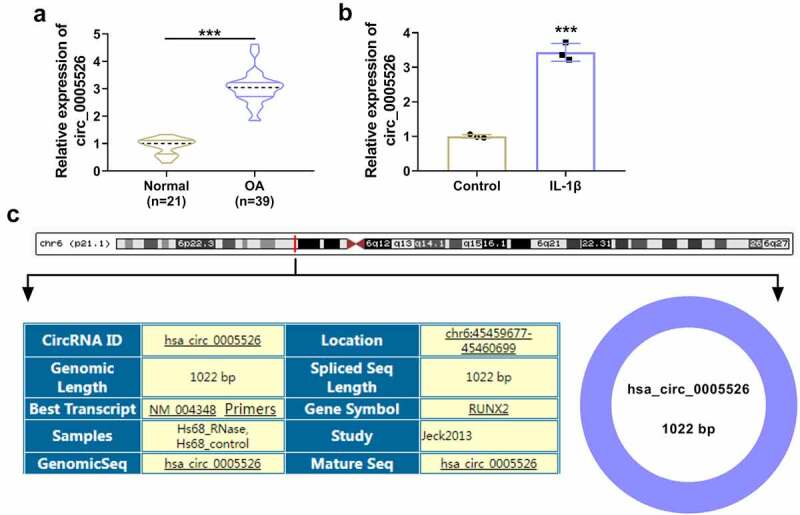
Circ_0005526 was upregulated in OA. (a-b) RT-qPCR was used for expression detection of circ_0005526 in OA and normal cartilage tissues (a), as well as 10 ng/mL IL-1β-treated CHON-001 cells and control cells (b). (c) Genic information of circ_0005526. ***P < 0.001.

CircRNA dysregulation is associated with OA progression and circRNA function is related to miRNA/mRNA network. The aim of this research was to investigate functional mechanism of circ_0005526 in OA. Circ_0005526 was considered to have regulatory effect on TCF4 by targeting miR-142-5p. Firstly, expression analysis of circ_0005526 was conducted by RT-qPCR. Relative to normal cartilage tissues and control cells, circ_0005526 level was significantly upregulated in OA cartilage tissues (Figure 1(a)) and 10 ng/mL IL-1β-treated CHON-001 cells (Figure 1(b)). Circ_0005526 is produced by back-splicing of runt-related transcription factor 2 (RUNX2) in chr6: 45459677–45460699with spliced sequence of 1022 bp (Figure 1(c)). The high expression of circ_0005526 implied the potential regulation in OA progression.

### IL-1β-induced inhibition of cell viability and proliferation but promotion of cell apoptosis and inflammation were relieved by circ_0005526 knockdown

**Figure 2.**
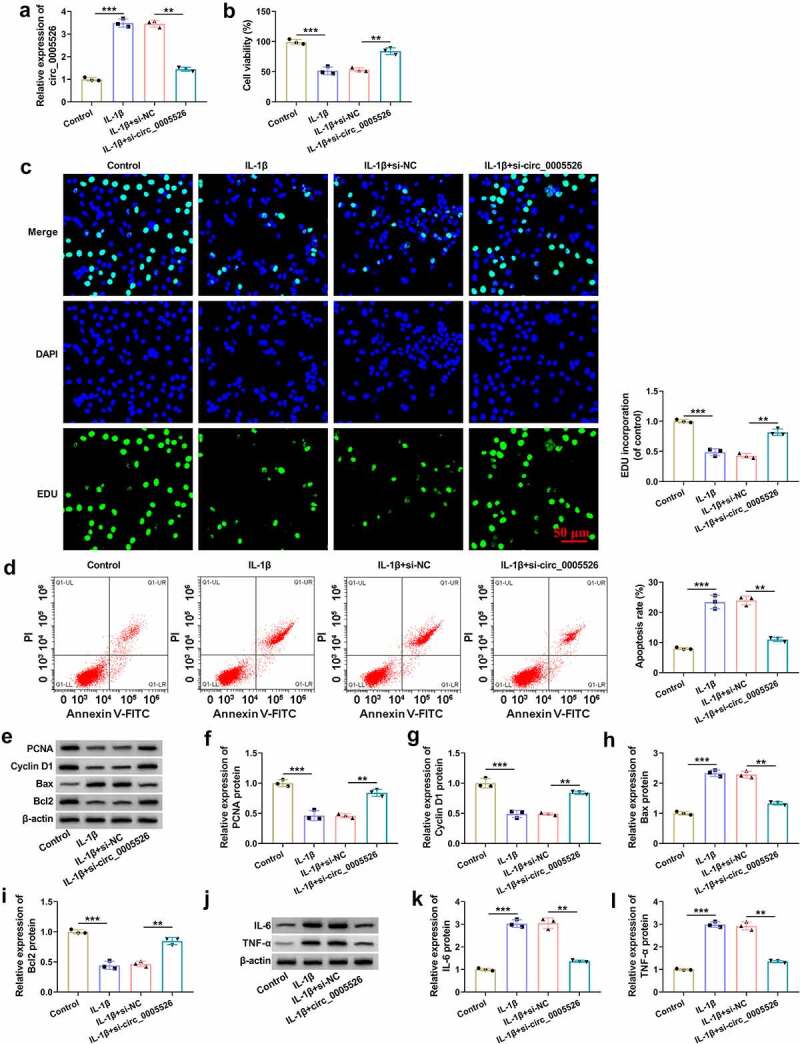
IL-1β-induced inhibition of cell viability and proliferation but promotion of cell apoptosis and inflammation were relieved by circ_0005526 knockdown. CHON-001 cells were treated with control, IL-1β (10 ng/mL), IL-1β+si-NC, or IL-1β+si-circ_0005526. (a) Circ_0005526 level analysis was conducted using RT-qPCR. (b) Cell viability detection was conducted using CCK-8 assay. (c) Cell proliferation examination was conducted using EdU assay. (d) Cell apoptosis evaluation was conducted via flow cytometry. (e-i) PCNA, Cyclin D1, Bax and Bcl2 protein determination was conducted via western blot. (j-l) IL-6 and TNF-α protein quantification was conducted by western blot. **P < 0.01, ***P < 0.001.

IL-1β-treated CHON-001 cells were transfected with si-circ_0005526 or si-NC to investigate circ_0005526 function in OA. The transfection efficiency was assessed using RT-qPCR. As shown in Figure 2(a), IL-1β-induced upregulation of circ_0005526 was significantly attenuated by si-circ_0005526. Cell viability by CCK-8 assay (Figure 2(b)) and cell proliferation by EdU assay (Figure 2(c)) were reduced by treatment of IL-1β, then si-circ_0005526 partly eliminated these effects. The result of flow cytometry revealed that cell apoptosis rate was decreased in IL-1β+si-circ_0005526 group compared to IL-1β+si-NC group (Figure 2(d)). The protein detection by western blot (Figure 2(e)) demonstrated that circ_0005526 inhibition reversed IL-1β-mediated downregulation of PCNA, Cyclin D1 and Bcl2, as well as upregulation of Bax in CHON-001 cells (Figure 2(f-i)). IL-6 and TNF-α protein levels were elevated after exposure to IL-1β, which was obviously restored by downregulation of circ_0005526 (Figure 2(j-l)). Overall, circ_0005526 knockdown inhibited IL-1β-induced chondrocyte injury.

### Circ_0005526 interacted with miR-142-5p

**Figure 3.**
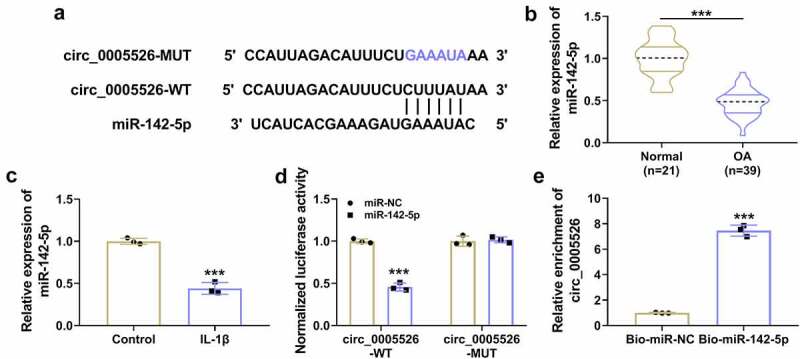
Circ_0005526 interacted with miR-142-5p. (a) Circinteractome showed the binding sequences between circ_0005526 and miR-142-5p. (b-c) The miR-142-5p level was detected via RT-qPCR in OA tissues (c) and CHON-001 cells exposed to 10 ng/mL IL-1β (C). (d-e) Dual-luciferase reporter assay (d) and RNA pull-down assay (e) were used for identification of circ_0005526 and miR-142-5p binding. ***P < 0.001.

Circinteractome (https://circinteractome.nia.nih.gov) indicated that circ_0005526 sequence contained miR-142-5p binding sites (Figure 3(a)). RT-qPCR data showed that miR-142-5p expression was downregulated in OA tissues compared with normal tissues (Figure 3(b)), and miR-142-5p downregulation was detected in IL-1β-treated CHON-001 cells relative to control group (Figure 3(c)). Transfection of miR-142-5p (compared to miR-NC transfection) reduced the luciferase activity in circ_0005526-WT group, but no significant change of luciferase activity was noticed in circ_0005526-MUT group (Figure 3(d)). Through performing RNA pull-down assay, we found that circ_0005526 could be pulled down by miR-142-5p in CHON-001 cells (Figure 3(e)). These evidences suggested that circ_0005526 directly interacted with miR-142-5p.

### The si-circ_0005526-induced protection was counteracted by inhibition of miR-142-5p in IL-1β-treated chondrocytes

**Figure 4.**
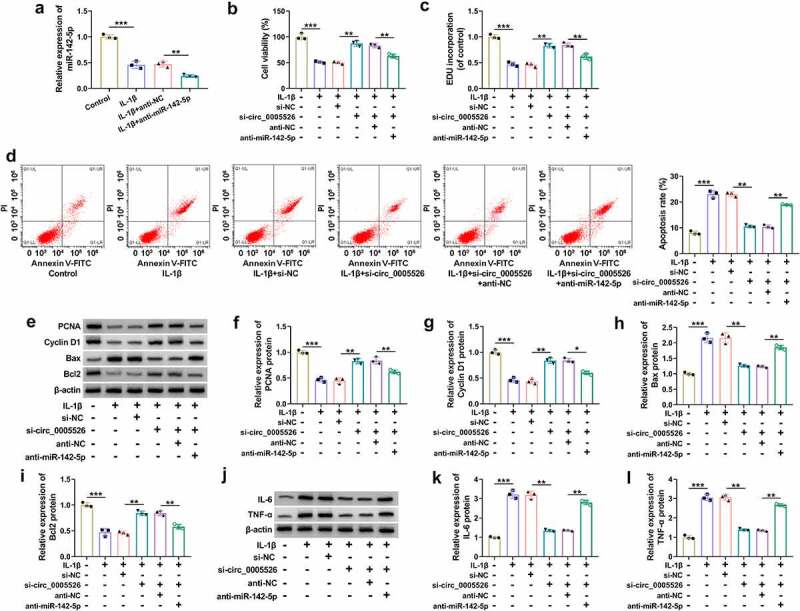
The si-circ_0005526-induced protection was counteracted by inhibition of miR-142-5p in IL-1β-treated chondrocytes. (a) RT-qPCR was applied for miR-142-5p quantification in control, IL-1β (10 ng/mL), IL-1β+anti-NC, or IL-1β+anti-miR-142-5p group. (b-l) CHON-001 cells were treated with control, IL-1β (10 ng/mL), IL-1β+si-NC, IL-1β+si-circ_0005526, IL-1β+si-circ_0005526+ anti-NC, or IL-1β+si-circ_0005526+ anti-miR-142-5p. (b) Cell viability was examined through CCK-8 assay. (c) Cell proliferation ability was assessed through EdU assay. (d) Apoptosis rate was determined via flow cytometry. (e-i) PCNA, Cyclin D1, Bax and Bcl2 protein levels were assayed by western blot. (j-l) IL-6 and TNF-α levels were detected using western blot. **P < 0.01, ***P < 0.001.

The relation of miR-142-5p with circ_0005526 function was further analyzed. The inhibitory efficiency of anti-miR-142-5p was excellent in IL-1β-treated CHON-001 cells (Figure 4(a)). The promoting effects of si-circ_0005526 on cell viability (Figure 4(b)) and proliferation ability (Figure 4(c)) were suppressed by anti-miR-142-5p in IL-1β-treated CHON-001 cells. Also, the protective function of si-circ_0005526 from IL-1β-induced cell apoptosis was abrogated by miR-142-5p inhibitor (Figure 4(d)). The si-circ_0005526-induced level changes in proliferation or apoptosis proteins (Figure 4(e-i)) and inflammatory markers (Figure 4(j-l)) were alleviated after miR-142-5p was downregulated in IL-1β-treated CHON-001 cells. Altogether, miR-142-5p sponging effect was responsible for the function of circ_0005526 in IL-1β-induced OA progression.

### Circ_0005526 regulated the level of TCF4 by targeting miR-142-5p

**Figure 5.**
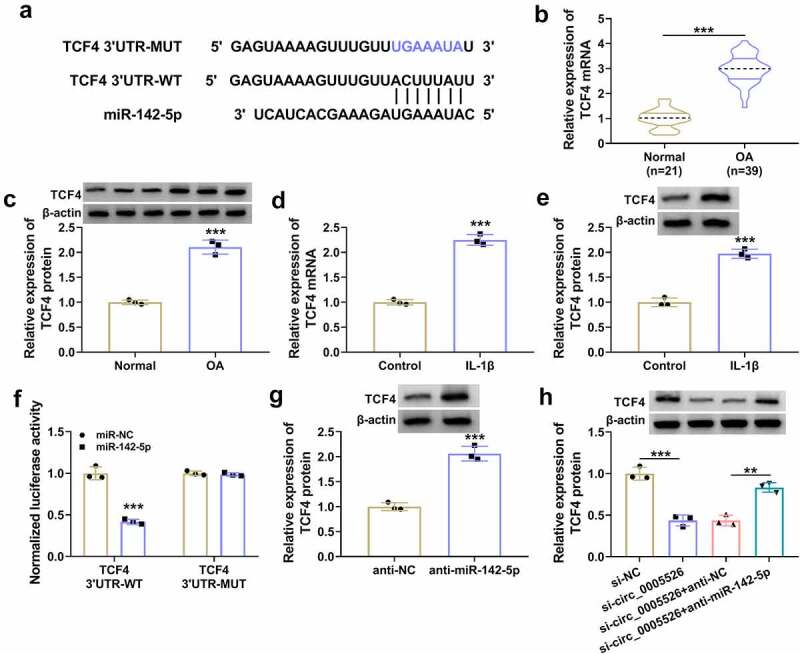
Circ_0005526 regulated the level of TCF4 by targeting miR-142-5p. (a) Targetscan exhibited the binding sites between miR-142-5p and TCF4 3ʹUTR. (b-e) TCF4 mRNA and protein levels were analyzed via RT-qPCR and western blot in OA tissues (b-c) and 10 ng/mL IL-1β-treated CHON-001 cells (d-e). (f) The binding analysis between TCF4 3ʹUTR and miR-142-5p was performed by dual-luciferase reporter assay. (g) TCF4 protein expression was determined after transfection of anti-NC or anti-miR-142-5p. (h) Western blot was applied for protein detection of TCF4 in si-circ_0005526, si-circ_0005526+ anti-miR-142-5p or the matched control groups. **P < 0.01, ***P < 0.001.

TCF4 3ʹUTR sequence was shown to have binding sites with miR-142-5p through prediction of Targetscan (http://www.targetscan.org) (Figure 5(a)). The mRNA and protein levels of TCF4 were markedly increased in OA tissues (Figure 5(b,c)) and IL-1β-treated CHON-001 cells (Figure 5(d,e)). Dual-luciferase reporter assay indicated that luciferase activity was inhibited by co-transfection with miR-142-5p and TCF4 3ʹUTR-WT, while this inhibition was not observed after co-transfection with miR-142-5p and TCF4 3ʹUTR-MUT in CHON-001 cells (Figure 5(f)). Furthermore, western blot displayed that TCF4 protein expression was upregulated by anti-miR-142-5p relative to anti-NC group (Figure 5(g)). Moreover, anti-miR-142-5p transfection mitigated TCF4 protein inhibition caused by si-circ_0005526 (Figure 5(h)). Thus, circ_0005526 could target miR-142-5p to regulate TCF4 expression.

### TCF4 overexpression reverted si-circ_0005526-induced effects in IL-1β-treated chondrocytes

**Figure 6.**
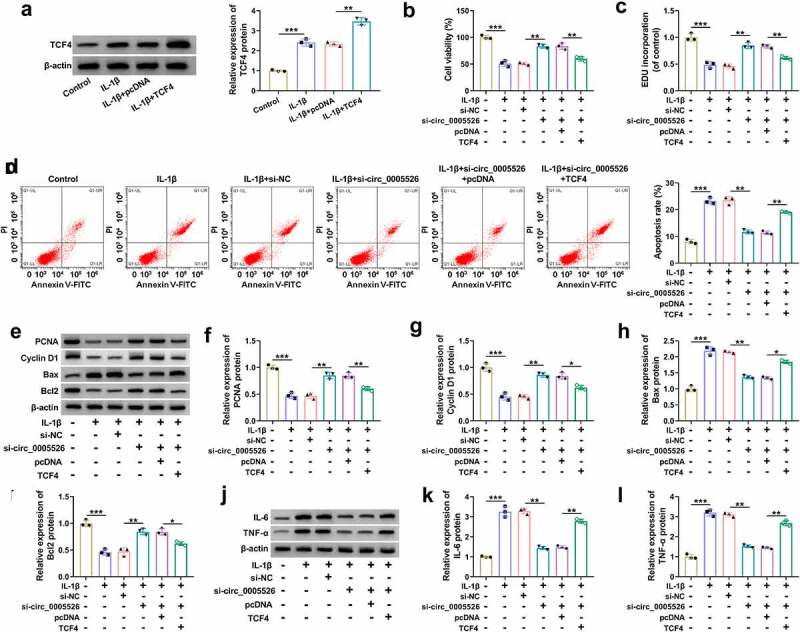
TCF4 overexpression reverted si-circ_0005526-induced effects in IL-1β-treated chondrocytes. (a) Western blot was performed to examine TCF4 protein level in control, IL-1β (10 ng/mL), IL-1β+pcDNA, and IL-1β+TCF4 groups. (b-l) CHON-001 cells were performed with treatment of control, IL-1β (10 ng/mL), IL-1β+si-NC, IL-1β+si-circ_0005526, IL-1β+si-circ_0005526+ pcDNA, or IL-1β+si-circ_0005526+ TCF4. (b) CCK-8 assay was performed to determine cell viability. (c) EdU assay was performed to assess cell proliferation. (d) Flow cytometry was performed to analyze cell apoptosis. (e-i) Western blot was performed to detect protein markers associated with proliferation and apoptosis. (j-l) Western blot was applied for level analysis of inflammatory proteins. *P < 0.05, **P < 0.01, ***P < 0.001.

Subsequently, a series of experiments were performed to research whether the role of circ_0005526 was related to TCF4. IL-1β-caused upregulation of TCF4 protein level was aggravated after transfection of TCF4, indicating the great overexpression efficacy of TCF4 (Figure 6(a)). Cell viability (Figure 6(b)) and proliferation (Figure 6(c)) were suppressed while apoptosis rate was enhanced (Figure 6(d)) in IL-1β+si-circ_0005526+ TCF4 group contrasted to IL-1β+si-circ_0005526+ pcDNA group. The regulatory effects of si-circ_0005526 on PCNA, Cyclin D1, Bax and Bcl2 were all offset after TCF4 overexpression in IL-1β-exposed CHON-001 cells (Figure 6(e-i)). In addition, IL-6 and TNF-α protein downregulation induced by si-circ_0005526 was countervailed by TCF4 in IL-1β-treated CHON-001 cells (Figure 6(j-l)). All in all, circ_0005526 regulated IL-1β-induced chondrocyte injury via upregulating TCF4.

## Discussion

The previous study indicated that circ_0005526 was upregulated in serum of OA patients, and had clinical significance in OA [10]. Our evidences unraveled that circ_0005526 knockdown inhibited chondrocyte injury in IL-1β-induced OA model through mediating miR-142-5p/TCF4 axis. Summary of the article search process was shown in Supplementary Fig. 1.

CircRNAs are identified to be associated with the pathogenesis of OA [22]. Hsa_circ_0045714 level downregulation reduced IL-1β-evoked OA chondrocyte injury [23]. Silencing circ_0001846 alleviated cell apoptosis and inflammation in IL-1β-induced OA progression [24]. CircRNA_0001236 contributed to chondrogenesis and inhibited cartilage degradation in OA [25]. Thus, circRNAs paly different roles in the development of OA. In the current research, circ_0005526 overexpression was detected in cartilage tissues from OA patients. Furthermore, circ_0005526 elevated cell viability and proliferation ability but suppressed cell apoptosis and inflammatory reaction in IL-1β-established OA model. These findings implied that circ_0005526 aggravated the progression of OA *in vitro*.

CircRNAs are implicated in disease development via sponging miRNAs. For instance, circUBE2J2 reduced migration and proliferation of hepatocellular carcinoma cells via controlling miR-370-5p [26]. CircRNA ubiquitin-specific protease 34 (circUSP34) exacerbated cell malignant behaviors of osteosarcoma by inhibiting miR-16-5p [27]. CircRNA-09505 enhanced inflammatory reaction in rheumatoid arthritis through absorbing miR-6089 [28]. Also, circRNA-Atp9b promoted extracellular matrix catabolism via targeting miR-138-5p in OA [29]. Circ_0005526/miR-142-5p interaction was confirmed in this study. In addition, miR-142-5p downregulation eliminated the regulatory function of circ_0005526 knockdown in IL-1β-induced OA injury. Hence, circ_0005526 facilitated OA progression through sequestering miR-142-5p in part.

The ceRNA hypothesis is defined that non-coding RNAs can downregulate gene expression via suppressing miRNAs binding to downstream mRNAs [30]. CircRNA/miRNA/mRNA axis was also uncovered in joint diseases. Circ_0088194 enhanced cell migration and invasion in rheumatoid arthritis through reducing the inhibitory effect of miR-766-3p on matrix metalloproteinases 2 (MMP2) [31]. Zhong *et al*. disclosed that circ_0088036 accelerated the progression of rheumatoid arthritis via miR-140-3p-related sirtuin-1 (SIRT1) upregulation [32]. Additionally, circ_0134111 promoted OA chondrocyte damage by inhibiting miR-224-5p to induce overexpression of CC motif ligand 1 (CCL1) [33]. Circ_0045714 increased phosphoinositide-3-kinase regulatory subunit 1 (PIK3R1) level to regulate IL-1β-stimulated chondrocyte injury via serving as a ceRNA of miR-331-3p [23]. In this study, miR-142-5p directly targeted TCF4 and circ_0005526 led to upregulation of TCF4 expression via targeting miR-142-5p. More importantly, siRNA circ_0005526-mediated protection against IL-1β-induced OA injury was suppressed by TCF4 upregulation. In other words, circ_0005526 regulated OA progression via upregulating the level of TCF4. The issued studies in Bioengineered also showed that circRNAs affected OA development by targeting miRNA/mRNA axis, such as circ_0005567/miR-492/SOCS2 and ciRS-7/miR-7/ IL-17A [34,35]. For the first time, circ_0005526 was considered to participate in OA progression through targeting miR-142-5p/TCF4 network.

This study still has some limitations. For example, the results were acquired from cell experiments. There is no data from *in vivo* model due to the limited condition and fund. The further exploration *in vivo* may be performed in future study. In addition, circRNAs can act on many miRNAs and miRNAs can target different genes. It is necessary to explore whether circ_0005526 function in OA is related to other miRNA/mRNA axes.

## Conclusion

**Figure 7.**
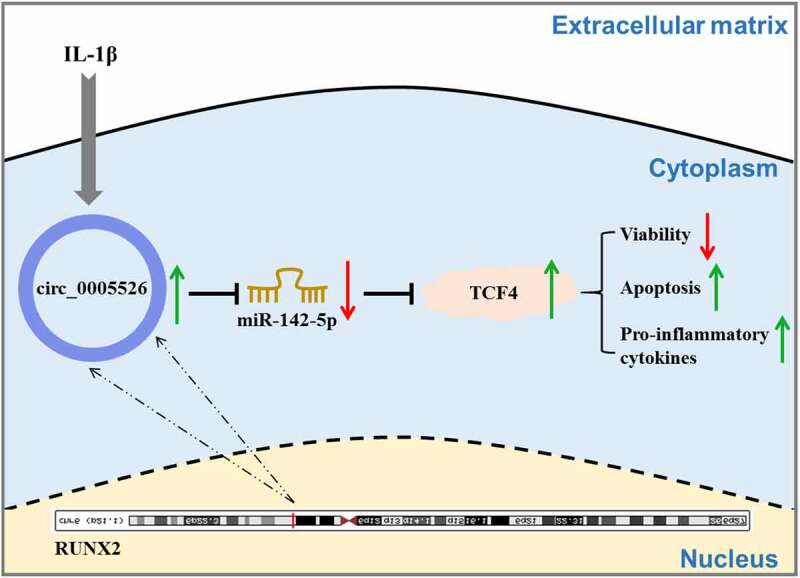
Graphical abstract of this study.

In conclusion, circ_0005526 aggravated IL-1β-incurred chondrocyte dysfunction via inhibiting miR-142-5p to upregulate TCF4 (Figure 7). This study elucidated a novel molecular pathogenesis circ_0005526/miR-142-5p/TCF4 in OA progression.

## Supplementary Material

Supplemental Material
