# Supplementary figures and images for: Circ_0005526 contributes to interleukin-1β-induced chondrocyte injury in osteoarthritis via upregulating transcription factor 4 by interacting with miR-142-5p
Source: Bioengineered. 2022 Mar 24;13(4):8407–18. doi: 10.1080/21655979.2022.2048773 (PMC9162016; doi:10.1080/21655979.2022.2048773)

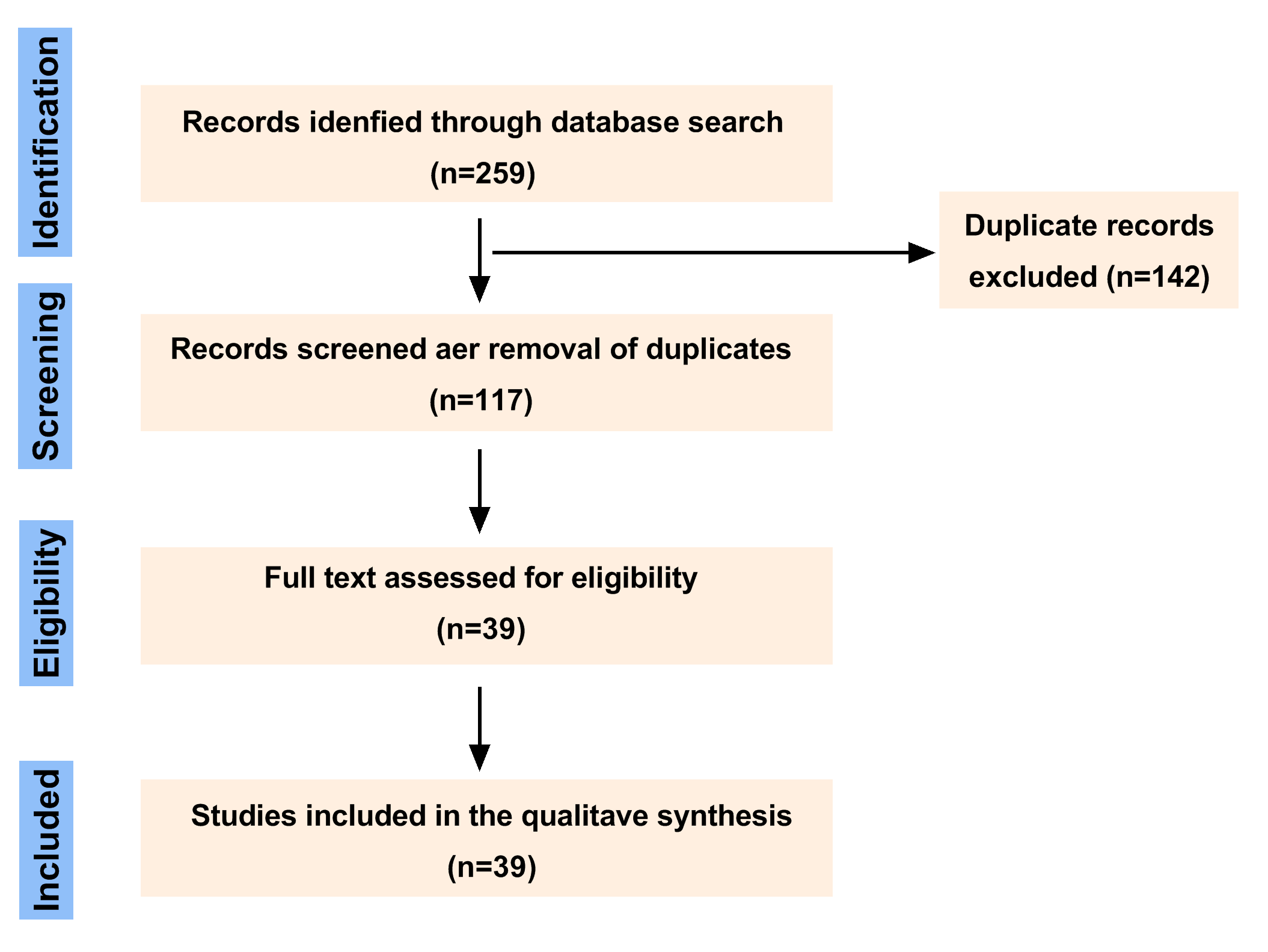

Supplement: Supplemental Material [file KBIE_A_2048773_SM9318.tif]
